# Impacts of Warming, Acidification, and Deoxygenation on Embryos and Larvae of Gilthead Seabream (*Sparus aurata*)

**DOI:** 10.3390/biology15131068

**Published:** 2026-07-03

**Authors:** Marta S. Pimentel, Catarina P. Santos, Maria R. Pegado, Eduardo Sampaio, Pedro Pousão-Ferreira, Vanessa M. Lopes, David Abreu dos Santos, João Caramelo, Rui Rosa

**Affiliations:** 1 MARE—Marine and Environmental Sciences Centre/ARNET–Aquatic Research Network, Laboratório Marítimo da Guia, Faculdade de Ciências, Universidade de Lisboa, Av. Nossa Senhora do Cabo 939, 2750-374 Cascais, Portugal; cdcsantos@ciencias.ulisboa.pt (C.P.S.); mritapegado@gmail.com (M.R.P.); kuka.forbesi@gmail.com (V.M.L.); davidsantos226@gmail.com (D.A.d.S.); joaocaramelo1999@hotmail.com (J.C.); rrosa@fc.ul.pt (R.R.); 2 Department of Animal Biology, Faculty of Science, University of Lisbon, 1749-016 Lisbon, Portugal; 3 Department of Collective Behaviour, Centre for the Advanced Study of Collective Behaviour, University of Konstanz, 78464 Konstanz, Germany; esampaio@ab.mpg.de; 4 Department of Animal Biology, University of Konstanz, 78464 Konstanz, Germany; 5 Instituto Português do Mar e da Atmosfera, Av. 5 de Outubro s/n, 8700-305 Olhão, Portugal; pedro.pousao@ipma.pt

**Keywords:** fish embryos, larvae, multi-factorial, deadly-trio, physiology, behavior

## Abstract

The interaction between increased dissolved carbon dioxide, rising temperatures, and oxygen loss—the so-called “deadly trio”—is expected to strongly affect marine biota over the coming years, undermining ocean services and uses. Nonetheless, no study has so far scrutinized the cumulative impact of these three stressors on fish embryos and larvae. To fill this knowledge gap, we conducted a fully multi-factorial experiment to investigate the effects of warming (+4 °C: 22 °C), acidification (Δ − 0.4 pH units: 7.7 pH, *p*CO_2_ ~1000 μatm), and deoxygenation (Δ − 60% O_2_ saturation: 3 mg O_2_ L^−1^) on physiological and behavioral responses of the commercially important species *Sparus aurata*. Deoxygenation was the primary factor reducing hatching rates (64.25%), survival (46.71%), and heart rates (31.99%) of recently hatched larvae, being generally further exacerbated when combined with warming and acidification. No larvae exposed to the interaction of the three treatments reacted to the phototactic behavior test. However, acidification alone caused a 50% reduction in phototactic behavior. Our findings demonstrate that the deadly trio is detrimental to early fish development, impacting several key features at this critical life stage, and the need to assess the impacts of stressors’ interaction on marine taxa to better predict future ecosystem responses to ocean changes.

## Simple Summary

This study evaluated how the combination of ocean warming, acidification, and deoxygenation (“deadly trio”) affects the early development of the fish *Sparus aurata*. Embryos and recently hatched larvae were exposed to increased temperature (Δ + 4 °C; 22 °C), elevated CO_2_ levels (*p*CO_2_ ~1000 μatm, Δ − 0.4 units; pH 7.7), and reduced oxygen (Δ − 60% O_2_ saturation; 3 mg O_2_ L ^−1^). Deoxygenation emerged as the primary stressor, significantly reducing hatching rates, larval survival, and heart rates. These effects were further intensified when combined with warming and acidification. Acidification alone also reduced larval phototactic behavior by 50%, while exposure to all three stressors eliminated phototactic responses entirely. Overall, the results demonstrate that multiple climate-related stressors together severely harm fish early life stages, emphasizing the need to study combined environmental changes to better predict future impacts of climate change on marine fish populations and ecosystem functioning.

## 1. Introduction

The oceans play a key role in mitigating global climate change by absorbing excessive heat content and greenhouse gases (e.g., carbon dioxide, CO_2_) from the atmosphere. This continuous uptake is driving widespread ocean warming and acidification [1]. Concurrently, ocean warming is predicted to exacerbate the loss of oxygen in the oceans (deoxygenation) through reduced oxygen solubility, increased respiration rates and ocean stratification [2,3,4,5,6]. These future environmental changes are projected to induce both gradual long-term and extreme short-term changes in temperature, pH, and oxygen levels across marine ecosystems [1,7,8,9,10,11]. Studies assessing the effects of climate change stressors have shown that warming, acidification, and deoxygenation can negatively affect fish early life stages by altering survival, growth, metabolism, development, and behavior [12,13,14,15,16,17,18]. Nevertheless, responses of fish early life stages to ocean climate changes have been shown to be highly variable, and also include neutral or weak effects in some species and contexts, highlighting strong species and trait dependencies rather than uniform negative outcomes, e.g., refs. [19,20,21,22]. Evidence from two-factor experiments indicates that these stressors rarely act independently, with warming–acidification, warming–deoxygenation, and acidification–deoxygenation interactions producing additive, synergistic, or antagonistic responses depending on the species and trait examined [23,24,25,26,27,28,29,30,31,32,33]. However, relatively few studies have evaluated the combined effects of deoxygenation with warming and/or acidification [34,35,36,37]. Meta-analyses and multi-stressor syntheses further demonstrate that interaction outcomes are often unpredictable from single-stressor responses alone and that the inclusion of additional stressors can generate emergent effects not detected in pairwise experiments [13,20,37,38,39]. Consequently, two-factor studies have substantially advanced our understanding of climate change impacts on marine fishes, but they remain insufficient to predict responses under future ocean conditions where warming, acidification, and deoxygenation will occur simultaneously, particularly during sensitive early developmental stages.

The combined and cumulative effects of warming, acidification, and deoxygenation, commonly referred to as the “deadly trio”, are expected to profoundly impact marine organisms by disrupting key physiological processes and amplifying the consequences of ongoing climate change [40,41,42,43,44,45,46]. These impacts may also propagate to ecosystem services, with significant socio-economic implications for fisheries and global food security [47,48]. Despite representing a high-rank priority in the field [3,13,38,44], experimental studies simultaneously addressing warming, acidification, and deoxygenation remain scarce [41,42,43,44,45]. As such, it is critical to study the effects of their interaction, ideally under multi-factorial experimental contexts to better disentangle the stressor-specific and combined effects [10,40,44,46]. To date, no study has scrutinized the impact of the interaction of the “deadly trio” on biological and behavioral features of fish at early stages, embryos and larvae, known to be one of the survival and/or developmental bottlenecks regarding species’ climate change vulnerability, e.g., [14,15,16,17]. Incorporating all three environmental factors is crucial to determine if synergistic interactions arise when fish are exposed to all of them at the same time because these types of interactions are expected to occur more frequently when evaluating the combined effects of three factors rather than just two or individual variables [36,42,45].

As one of the most economically important fish species in the Mediterranean and eastern North Atlantic, supporting both fisheries and aquaculture, the gilthead seabream (*Sparus aurata*) is a coastal and estuarine species that inhabits shallow habitats highly exposed to future environmental changes, namely warming, acidification, and episodic hypoxia [49,50]. Spawning in *Sparus aurata* occurs primarily in coastal waters during autumn and winter, and the early life stages develop as pelagic eggs and larvae within the highly variable surface layer of the water column before recruiting to coastal nursery habitats [51]. Throughout this period, individuals may experience substantial fluctuations in temperature, oxygen availability, and carbonate chemistry, making them particularly susceptible to simultaneous exposure to multiple climate-change-related stressors [3,8]. Previous studies have shown that embryos and larvae of this species are highly sensitive to warming and acidification, which negatively affect multiple performance traits [27,28]. Alongside this, later life stages also exhibit reduced performance under elevated temperatures and low-oxygen conditions, e.g., ref. [52]. These characteristics make *S. aurata* an ecologically relevant model for assessing the effects of concurrent exposure to the “deadly trio” during early development. In this context, the aim of the present study was to evaluate experimentally, for the first time, the impact of warming, acidification, and deoxygenation interactions on the early development of gilthead seabream *Sparus aurata*, namely on hatching rates, early survival, heart rates, body malformations, larvae responsiveness, and phototaxis rates.

## 2. Materials and Methods

### 2.1. Experimental Design and Egg Incubation

*Sparus aurata* eggs were collected immediately after spawning in April 2019 from adult wild-caught broodstock reared at Instituto Português do Mar e da Atmosfera (IPMA)—Centro Regional de Investigação Pesqueira do Sul (CRIPSul, Olhão, Portugal). After collection, eggs from the same batch were transferred to the aquaculture facilities in Laboratório Marítimo da Guia (Cascais, Portugal) and acclimated to different experimental conditions. Eggs and hatched larvae were exposed in a cross-factorial design to represent the deadly trio conditions [1] characterized by elevated temperatures (warming, W), decreased pH (acidification, A) and oxygen levels (deoxygenation, D), relative to control environmental conditions (see Table 1) during the natural spawning season [51,53]. As such, eggs were reared until hatching (~48 h), and recently hatched larvae were reared until two days after hatching at a density of 70 larvae L^−1^ [28] under a total of eight experimental treatments featuring the different combinations of stressors (Table 1).

Each replicate (three replicates per treatment) consisted of 20 L of sealed cylindrical experimental glass containers, placed in temperature-controlled water baths (one for the control and another for warmer temperatures), with chillers and heaters to maintain the desired temperature according to the experimental treatment. Inside each replicate, a smaller rearing box (each with 300 mL) laterally surrounded by a fine mesh size net of 100 μm was placed to monitor and account for egg hatching rates and larval survival. Treatment conditions, temperature (+4 °C; 22 °C), CO_2_ (Δ − 0.4 pH units: 7.7 pH, *p*CO_2_ ~1000 μatm) and O_2_ (Δ − 60% O_2_ saturation, ~3 mg O_2_ L^−1^) levels were automatically monitored and manually using multiparameter portable meters (VWR pH meter 1100H, and multimeter 3510 WTW GmbH; Weilheim, Germany). For each replicate, the designated treatment conditions were established in preconditioned tanks, where temperature, pH, and dissolved oxygen concentrations were continuously monitored and maintained at the target levels. Water quality was maintained through a gentle drip-flow system that continuously renewed the seawater in these preconditioned tanks, replacing approximately 10% of the total tank volume per day. The pH and O_2_ levels for each treatment condition were maintained through the addition of preconditioned seawater, adjusted in preconditioned tanks through the carefully monitored injection of CO_2_ and N_2_ certified gas mixtures and/or by aeration with CO_2_ filtered air, via air stones, in sealed glass tanks (20 L) sharing the same water bath as the corresponding treatment. Before entering the experimental tanks, the water was already preconditioned for each treatment and homogenized. Salinity was kept stable at ~35 (V2 Refractometer, TMC Iberia, Lisbon, Portugal), and the photoperiod was set to 14 L:10 D. This setup and adjustment intervals were defined after several preliminary experiments to ensure that targeted treatment conditions were maintained over the course of the experiment.

The experimental systems were equipped with biological filters (bioballs, ouriço^®^, Fernando Ribeiro Lda, Queluz, Portugal), protein skimmers (ReefSkimPro 400, TMC-Iberia, Lisbon, Portugal), and UV-sterilizers (Vecton 600, TMC Iberia, Lisbon, Portugal). Additionally, natural seawater UV-sterilized (Vecton 600, TMC Iberia, Lisbon, Portugal) and 0.35 μm filtered (Harmsco, Riviera Beach, FL, USA) was continuously renewed with a soft water drip system to the preconditioned tanks (around 10% of the total volume). Ammonia and nitrite were monitored regularly and kept below detectable thresholds. Total alkalinity was measured spectrophotometrically at 595 nm according to [54], and seawater carbonate chemistry (see Table 1) data for each treatment conditions was quantified by analyzing total carbon (C_T_), carbon dioxide partial pressure (*p*CO_2_), bicarbonate concentration (HCO_3^−^_) and aragonite saturation state of seawater (Ω_arag_) with CO2SYS software [55] using salinity, temperature, pH, and total alkalinity (A_T_).

### 2.2. Hatching Rates and Larvae Survival

To analyze *S. aurata* embryonic development, 10 eggs were randomly placed and reared inside the smaller rearing boxes (i.e., three boxes per treatment). Hatching rates were analyzed based on the number of hatched larvae from these eggs. Survival rates of the hatched larvae were determined by calculating the average of the three replicates per treatment, based on the number of surviving larvae two days after hatching.

### 2.3. Routine Heart Rates

Ten newly-hatched larvae, two days after hatching, collected from each replicate (*n* = 30 per treatment) were placed in sealed respirometry chambers, filled with seawater from the respective treatment (according to [28]). Water volumes were adjusted to allow routine activity and to minimize larval stress. Routine heart rate measurements were taken at the end of the experiment under a stereoscope (Leica S6D, Leica Microsystems, Wetzlar, Germany), and defined as the number of heart beats per unit of time, when larvae were not swimming (according to [28]).

### 2.4. Body Malformations

Two days after hatching, 10 *S. aurata* larvae were collected from each replicate (*n* = 30 per treatment) and observed under a microscope to quantify body structure malformations and/or axial deviations, e.g., abnormal body curvatures such as side-to-side, excessive inward or outward curvatures, based on [21,27,56]. Malformation rates were quantified as the percentage of fish exhibiting a specific deformity in their body structure. The observer was not aware of the treatment provenance of each larva.

### 2.5. Phototaxis

Larvae two days after hatching responsiveness to light stimulus, as well as positive (swimming towards a light source) or negative (swimming away from a light source) phototaxis, were tested individually in 3 newly-hatched *S. aurata* larvae per replicate (*n* = 9 per treatment) inside a 30 cm long glass swim channel containing water from each treatment, with a source of light (LED) at one end adapted from [57]. During the test, the swimming channel was covered to reduce the influence of outside light. Larvae were placed in a “start compartment” in the middle of the raceway, and after the separating gate was removed, larvae could move either to the light source or away from it. Each larva was randomly selected from the original tank and allowed to acclimate behind the removable gate. After this period, the gate was lifted, and the proportion of larvae that (1) moved towards the light source side (LED), (2) moved away from the light source or (3) had no response after 2 min (i.e., larvae stayed in the starting area without moving) was registered. This test yielded two response variables: larval response rates (leaving the arena or not moving) and positive or negative phototactic responses (moving towards the light or away from the light, respectively). Larval phototaxis was quantified as the percentage of fish exhibiting positive phototaxis related to the total number of larvae that exhibited both positive and negative phototaxis. Phototactic response was observed through direct visual observation by a single observer who was blind to the experimental treatments.

### 2.6. Statistical Analysis

Statistical analyses of all the defined variables were performed with RStudio Software (Version 1.3.959 © 2009–2020 RStudio, Inc., Boston, MA, USA, 2020). All data (i.e., hatching rates, survival, heart rates, malformations, response rates to the phototactic test, and phototactic response) were first analyzed univariately via generalized linear mixed models (GLMM, e.g., ref. [58]) with all the environmental stressors as factors (see model families used in Table S1). Following recommendations from [59], all replicates (tanks and also boxes for hatching rates) of each variable analyzed were included and kept in GLMM models as random effects to account for potential variability in the experimental design, independently of the amount of variation explained. All GLMM assumptions (independence, normality, and homoscedasticity of residuals) were tested. The package “check_model” was used to validate the models’ performance and assumptions. When significant interactions between factors were found, all post hoc pairwise comparisons between groups were performed using the R package *emmeans* [60].

### 2.7. Ethical Statement

This study was approved by the Portuguese National Science Foundation (FCT), the FCUL Animal Welfare Committee (ORBEA FCUL) and the Ethical Committee of the Portuguese General Veterinary Directorate (DGAV) in accordance with National (Decreto-Lei 113/2013) and EU legislation (Directive 2010/63/EU) on the protection of animals used for scientific purposes. The Laboratory Marίtimo da Guia, MARE-ULisbon has also obtained the certification No. PT03200OCI from TRACES and DGAV for the use of laboratory animals to meet scientific research needs.

## 3. Results

### 3.1. Hatching Rates and Survival

**Figure 1:**
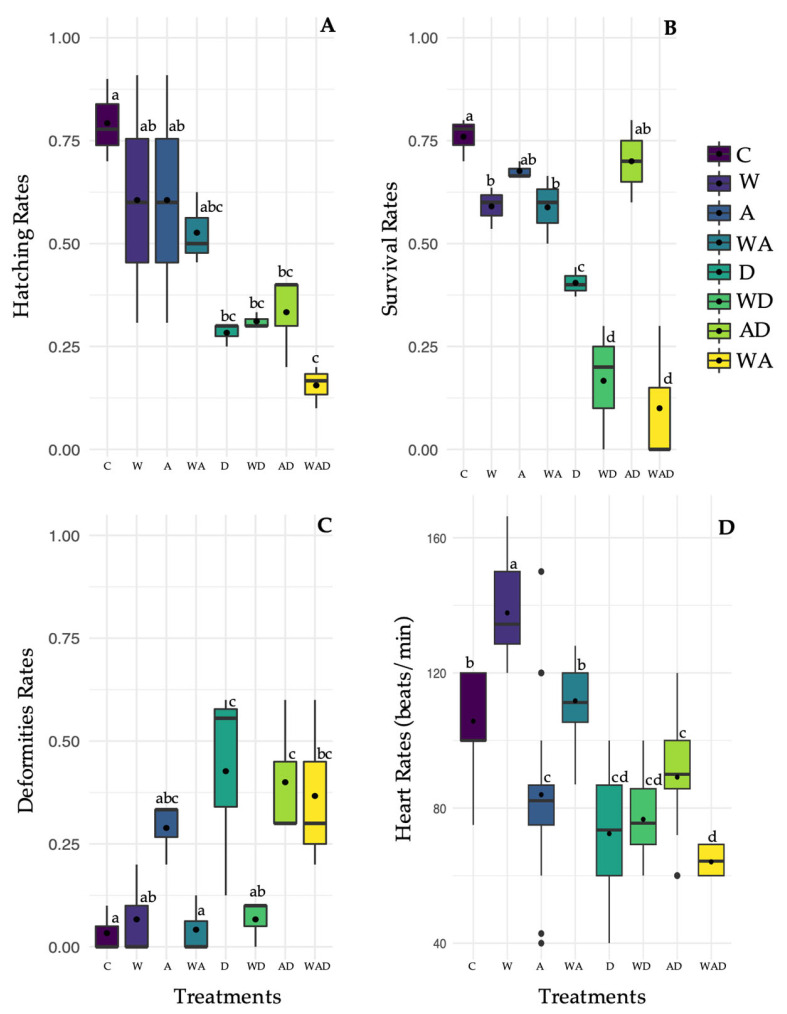
Effect of warming, acidification, and deoxygenation on the hatching rates (**A**), survival rates (**B**), deformity rates (**C**) and routine heart rates (**D**) of early developmental stages of *S. aurata*. Statistical differences (α = 0.05) between treatments are represented by different superscript letters over each plot. Legend description: C—control, W—warming, A—acidification, W + A—warming + acidification, D—deoxygenation, W + D—warming + deoxygenation, A + D—acidification + deoxygenation, and W + A + D—warming + acidification + deoxygenation experimental treatment. Different lowercase letters indicate statistically significant differences among groups (*p* < 0.05).

Hatching rates of *S. aurata* showed a decreasing trend after exposure to warming and acidification (Figure 1A, see also Table S1); however, deoxygenation was the only isolated factor to trigger a significant and drastic reduction in hatching rates (GLMM analysis, binomial family, *p* < 0.001; Table S1).

From control values, hatching rates significantly decreased by 64.25% after eggs were exposed to isolated deoxygenation, 57.94% after exposure to acidification combined with deoxygenation, and 60.75% after exposure to the combination of warming with deoxygenation (multi-treatment post hoc comparative analysis, D: *p* < 0.001, A + D: *p* = 0.0014, W + D: *p* = 0.0006, respectively). However, no significant differences were found between these three treatments. On the other hand, the exposure to the combined effect of warming, acidification and deoxygenation induced an even more accentuated and significant decrease of nearly 80.37% to a hatch rate of 15.56 ± 5.09% (multi-treatment post hoc comparative analysis, W + A + D: *p* < 0.0001).

Larvae survival (Figure 1B) significantly decreased from control conditions (75.95 ± 5.27%, Figure 1B) after exposure to acidification, warming and deoxygenation, as single factors (GLMM analysis, binomial family, A: *p* < 0.01, W: *p* < 0.001, D: *p* < 0.001; Table S1), as well as under all combined treatments that included deoxygenation (GLMM analysis, binomial family, A + D: *p* < 0.001, W + D: *p* < 0.05; W + A + D: *p* < 0.001; Table S1). However, post hoc pairwise comparisons of marginal means revealed no significant differences between control and the combination of acidification with deoxygenation (estimate = 0.05960, SE = 0.0306, *z* = 1.946, *p* = 0.5189).

The combination of warming with deoxygenation, and the triple combination led to the lowest survival rates observed, namely 16.67 ± 15.28% and 10 ± 17.32%, respectively (multi-treatment post hoc comparative analysis, W + D: *p* < 0.0001, W + A + D: *p* < 0.0001). No significant differences were detected between these two experimental treatments (multi-treatment post hoc comparative analysis, *p* = 0.0876).

### 3.2. Body Malformations

Regarding *S. aurata* early developmental malformations (Figure 1C), both acidification and deoxygenation, as single factors, induced a significant increase in their incidences (GLMM analysis, binomial family, A: *p* = 0.034 and D: *p* = 0.004; Table S1). The interaction of deoxygenation with all the other treatments also induced significant increments in the rate of malformations (GLMM analysis, binomial family, A + D: *p* = 0.039, W + D: *p* = 0.036 and W + A + D: *p* = 0.008; Table S1). However, post hoc pairwise comparisons of marginal means revealed no significant differences between control and the combination of warming with deoxygenation (estimate = −0.164, SE = 0.284, z = −0.577, *p* = 0.9991). The incidence of malformations of recently hatched larvae increased from 3.33 ± 5.77% at control conditions to 36.67 ± 20.82%, 40.00 ± 17.32%, and to 42.68 ± 26.24% after exposure to the combination of the three factors, the combination of acidification with deoxygenation, and deoxygenation as a single factor, respectively (multi-treatment post hoc comparative analysis, W + A + D: *p* = 0.0092, A + D: *p* = 0.003, D: *p* = 0.0012). Although no significant differences were detected among these three conditions, deoxygenation alone induced the highest percentage of deformities.

### 3.3. Routine Heart Rates

Exposure to warming, acidification and deoxygenation, as single factors, prompted significant changes in the routine heart rates of recently hatched *S. aurata* larvae (GLMM analysis, Gaussian family, *p* < 0.001 for the three factors; Table S1).

Moreover, heart rates were also significantly affected by the interaction of deoxygenation with all the other treatments (GLMM analysis, binomial family, A + D: *p* < 0.001, W + D: *p* < 0.001, W + A + D: *p* < 0.05; Table S1). While the combination of warming with acidification did not differ from the control (*p* = 0.937), treatments with deoxygenation resulted in a significant reduction in heart rates (*p* < 0.0001). Routine heart rates increased significantly under warming, whereas acidification as a single factor triggered a significant decrease (multi-treatment post hoc comparative analysis, W: *p* < 0.0001, A: *p* = 0.0001). However, post hoc pairwise comparisons of marginal means revealed that the warming-induced increase was no longer observed when warming was combined with acidification or deoxygenation, as both combinations resulted in significantly lower heart rates relative to the warming treatment (estimate = 25.83 and 60.81, SE = 5.30 and 5.90, df = 143 and 143, and *t* = 4.874 and 10.300, and *p* = <0.0001 and <0.0001, respectively).

With the exception of the interaction between warming and acidification, all the remaining treatments induced a significant decrease in heart rates (multi-treatment post hoc comparative analysis, W + A: *p* = 0.937, D: *p* < 0.0001, W + D: *p* < 0.0001, A + D: *p* = 0.039). The combination of the three factors induced the highest significant decrease of 39.39%, from 105.71 ± 14.25 at control conditions to 64.07 ± 3.76 beats per minute (multi-treatment post hoc comparative analysis, *p* < 0.0001).

### 3.4. Response Rates and Phototaxis

**Figure 2:**
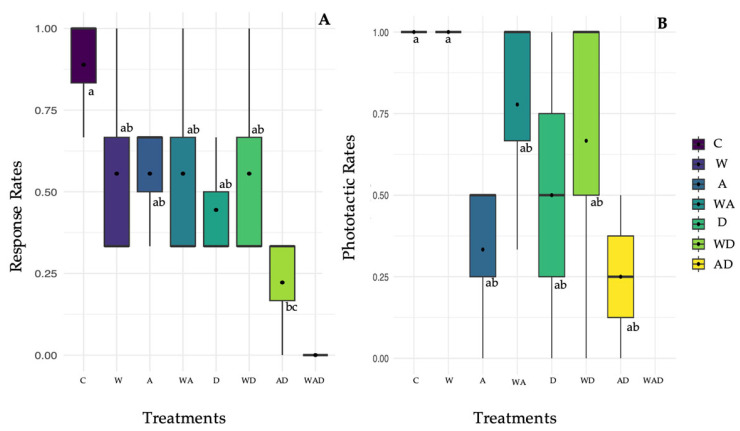
Effect of warming, acidification, and deoxygenation on the phototactic response of early developmental stages of *S. aurata*. Response rates during the phototactic test (**A**) and phototactic rates (**B**) of recently hatched larvae of *S. aurata* exposed to different experimental treatments. Legend description: C—control, W—warming, A—acidification, W + A—warming + acidification, D—deoxygenation, W + D—warming + deoxygenation, A + D—acidification + deoxygenation, and W + A + D—warming + acidification + deoxygenation experimental treatment. Different lowercase letters indicate statistically significant differences among groups (*p* < 0.05).

Although the exposure to the environmental stressors tested produced a decreasing trend in the proportion of recently hatched larvae that responded to the behavior test (Figure 2A) in relation to control conditions, no significant differences were detected (GLMM analysis, binomial family, *p* > 0.05; Table S1). However, pairwise comparisons of marginal means revealed that the control treatment differed from the AD and WAD treatment (estimate = 6.43 × 10^−1^ and 8.93 × 10^−1^, SE = 0.186 and 0.104, Z =3.457 and 8.571, *p* = 0.0128 and <0.0001, respectively), and that WAD differed from A, W, WA, WD (for all, estimate = 5.57 × 10^−1^, SE = 0.173, Z = 3.215, *p* = 0.0285). It is worth noting that none of the larvae exposed to the three-stress cumulative treatment (W + A + D) reacted to the phototactic behavior test (Figure 2A); consequently, no larvae presented a phototactic response (Figure 2B). Meanwhile, exposure to acidification resulted in a significant decrease in the proportion of phototactic larvae (Figure 2B) (GLMM analysis, Gaussian family, *p* = 0.042, Table S1). On the other hand, neither W, D, nor any of the interactions between factors induced a significant phototactic response (see Table S1). Post hoc pairwise comparisons of marginal means revealed that the control and W treatment differed from the WAD treatment (for both: estimate = 1.000, SE = 0.267, df = 15.8, *t.ratio* = 3.739, *p* = 0.0301).

## 4. Discussion

The present study demonstrates that early life stages of *Sparus aurata* are highly sensitive to future ocean change, although responses varied substantially across traits and stressor combinations. Consistent with previous studies reporting negative effects of warming, acidification, and deoxygenation on fish early development, e.g., [21,22,23,24,25,26,27,28,35], we found that the combined exposure to these stressors altered hatching success, survival, cardiac performance, developmental abnormalities, and phototactic behavior. However, the strength of these responses was not uniform throughout the analyzed biological endpoints. Although the three-stressor treatment generally produced the most pronounced effects, it was not consistently significantly more severe than those observed under specific two-stressor combinations. These findings highlight the importance of evaluating individual endpoints separately and suggest that the biological consequences of the “deadly trio” are shaped by complex interactions among stressors rather than by a universally stronger effect of simultaneous exposure.

Within our experimental setup, deoxygenation alone triggered a marked decrease (~64%) in hatching rates. This decrease was further exacerbated when deoxygenation was combined with both warming and acidification, reducing hatching success to 15.56 ± 10.08%, a nearly 45% decrease relative to the isolated effect of deoxygenation. The addition of warming and/or acidification may have contributed to relatively fewer additional stress-producing effects that were not statistically distinguishable from those caused by deoxygenation alone. This pattern suggests that oxygen limitation was the primary driver of the observed responses. When exposed to the “deadly trio” treatment, most embryos were not able to free their heads from the chorion, preventing successful hatching, which suggests that this process was more challenging under such conditions. Previous studies already showed that although the morphological characteristics of fish eggs provide embryos with some physical barrier and protection during exposure to environmentally stressful conditions, their protection may not be fully effective, undermining this critical hatching process, e.g., [24,35,61]. When subjected to environmental stressors, some changes in the metabolic pathways of fish can occur, e.g., [28,62,63,64], and ultimately may change the allocation of energy in the organisms. Although these metabolic shifts were not measured in the current study, we hypothesize that the combined stressors tested here may compromise fish metabolic and energetic pathways, potentially reducing energy availability for certain processes, particularly those that are energetically demanding, such as the hatching process, which is considered a very energetically expensive process [65,66].

Survival rates of recently hatched larvae were negatively affected by warming, warming combined with acidification, deoxygenation and deoxygenation combined with warming and acidification. Similar reductions in survival during early developmental stages have been reported in several fish species exposed to elevated temperature, ocean acidification, and deoxygenation [21,25,26,67,68,69], including *S. aurata* [27,28]. In the present study, with the exception of the pairwise combination of deoxygenation with acidification, treatments containing deoxygenation generally resulted in the lowest survival rates. The strongest reduction in survival was observed under the combined exposure to warming, acidification, and deoxygenation, with survival rates of newly hatched larvae decreasing by up to 86.83% relative to control conditions. Contrary to hatching rates, when deoxygenation was combined with acidification, no significant differences were found from the control or from acidification. This result suggests that the negative effects of deoxygenation on larval survival were not amplified by elevated CO_2_. Although the underlying mechanisms were not examined here, the absence of an additive effect of deoxygenation and acidification on larval survival may reflect stage-specific sensitivity and/or physiological compensation that reduced the impact of oxygen limitation after hatching.

As previously reported [28], the heart rates of *S. aurata* larvae increased with warming and decreased with acidification. The absence of the warming-induced increase in heart rate under the WA treatment, together with the reduction observed under WD relative to control conditions, suggests that acidification and deoxygenation may have counteracted the physiological stimulation induced by warming, resulting in a possible antagonistic multi-stressor response. Although the lowest heart rates were recorded under the combined exposure to warming, acidification, and deoxygenation, this treatment was not significantly different from deoxygenation alone or from deoxygenation combined with warming. Therefore, unlike what would be expected under a synergistic multi-stressor response, the addition of warming did not result in a statistically detectable reduction in heart rate beyond that caused by deoxygenation itself. These findings indicate that deoxygenation was the primary driver of the observed cardiac response, while the contribution of the additional stressors was comparatively limited.

Responses of fish early life stages to deoxygenation vary among species, with some exhibiting increased heart rates as a compensatory response [70], whereas others, as observed here, show marked reductions in cardiac activity [71,72,73]. The observed decrease in heart rate may reflect the limited physiological capacity of embryos and larvae to cope with reduced oxygen availability. At these developmental stages, cardiovascular and respiratory systems are still developing, constraining the ability to maintain oxygen uptake and transport under hypoxic conditions [74]. Consequently, oxygen limitation may impair aerobic metabolism and physiological performance, potentially contributing to the reduced survival observed in the present study [62,75,76,77,78].

Previous studies have detected an increment of deformities in fish larvae under single warming, acidification and deoxygenation exposure, e.g., [21,79,80,81], but few have analyzed the interactive effects of these stressful factors in its incidence, e.g., [25,27,33]. Acidification and deoxygenation elicited the highest deformity rates. Deoxygenation by itself also had a significant and comparatively greater effect on the incidence of early body malformations of recently hatched larvae. The incidence of malformations increased up to nearly 40% after exposure to deoxygenation, to its combination with acidification, and to the triple interaction. Contrary to hatching rate results, when deoxygenation was combined with warming, no significant differences were found from either the control or from warming. Deoxygenation might increase the frequency of malformations in fish early development, as shown for other species [82,83,84,85], because low oxygen possibly derails embryos’ normal development and triggers premature hatching when oxygen becomes limited before fish embryos are fully developed. High malformation rates in early developmental stages can affect fish feeding efficiency and swimming capabilities, and lately their survival [86], which can be problematic for fish populations. The resulting increase in developmental abnormalities can impair feeding, swimming performance, and ultimately survival, with important implications for recruitment and population dynamics, and over time, these changes may induce considerable socio-economic impacts.

Visually guided behaviors, like phototaxis, are fundamental behaviors that are closely related to fish environmental life conditions and the feeding habits of each fish species [87,88,89]. This behavior confers several benefits to many marine fishes [90,91], especially in the period immediately after hatching [90,92] by enabling larvae to start foraging, capture prey, and survive [90,91]. Response rates to the presence of a light stimulus, as well as making the correct decision (move towards or away from the light, i.e., positive or negative phototaxis), are key components in the ecology of phototactic fishes that can be impacted under future environmental conditions. Previous studies have shown that fish behavioral responses to visual stimuli, including phototaxis, can be affected by future climate change conditions, e.g., [93], such as acidification [57] and deoxygenation [94]. It was shown that high CO_2_ induced an increment in phototactic response of two-spotted goby (*Gobiusculus flavescens*) [57], and ref. [94] reported a deoxygenation-associated loss of phototactic response in walleye (*Sander vitreus*) fish species. The present results point in the same direction as the aforementioned study, with a potential effect of deoxygenation in decreasing responsiveness to light stimulus, especially when combined with acidification and both acidification and warming. However, we observed an acidification-driven effect on higher error in decision-making, choosing more often to move away from the light, i.e., negative phototaxis. The present findings suggest that *S. aurata*’s natural reaction to light, i.e., high reactivity towards high light intensities and colors as an adaptation to pelagic living and hunting near surface waters [89], might be impaired by acidification and potentially by deoxygenation. The absence of an appropriate response here described may be a consequence of a lower sensitivity to light caused by high CO_2_ levels; in contrast, hypersensitivity to light, such as the one described by [57], may lead to maladaptive responses to weak or absent signals [95].

While the present study demonstrates strong negative effects of warming, acidification and deoxygenation, previous studies have also reported that combined effects of warming and acidification can result in neutral, additive, or even antagonistic responses, with no significant impacts on survival, growth, or developmental traits of fish early life stages, e.g., refs. [18,19,20,21]. Ocean warming–acidification experiments represent the most frequently studied multistressor combination in the literature, e.g., refs. [17,20,37]. Although deoxygenation has been shown to have a negative effect when combined with warming or acidification, studies that also incorporate deoxygenation similarly report neutral or non-significant effects on survival, growth, and developmental traits, alongside additive or antagonistic interactions depending on species and exposure conditions, e.g., refs. [8,33,34,35,36,37,38,39,96,97,98,99]. Collectively, these contrasting findings suggest that the effects of multiple stressors cannot be generalized across species and that physiological tolerance thresholds, life-history traits, and environmental histories likely modulate organismal responses.

In the present study, deoxygenation consistently emerged as the dominant stressor, substantially reducing hatching success and larval survival and increasing developmental abnormalities, both alone and in combination with the other stressors. This pattern supports growing evidence that declining oxygen availability may represent a particularly critical component of future ocean change scenarios. It is important to recognize that multi-stressor responses are highly species- and context-dependent. Therefore, rather than suggesting that the “deadly trio” will universally affect all fish species in the same manner, the present results highlight deoxygenation as a key driver of vulnerability in marine fish early life stages and emphasize the need for further comparative studies across taxa.

## 5. Conclusions

Deoxygenation likely represents a primary driver of physiological disruption in early life stages of fish, given their high oxygen demand, and may further reduce their capacity to withstand concurrent thermal and acidification stress [98,99]. Even though the oxygen levels used here would mostly align with extreme events rather than long-term trends predicted for the end of the century, these events are expected to become increasingly frequent, as are marine heatwaves and acidification events, with events featuring these factors often overlapping [7,8,9,11,99]. Overall, these findings highlight the importance of deoxygenation within the “deadly trio” and underscore the need for further multistressor studies to better predict organismal and ecosystem responses in a changing ocean.

## Figures and Tables

**Table 1:** Seawater carbonate chemistry data for *S. aurata* larvae under different treatment conditions. Total carbon (C_T_), carbon dioxide partial pressure (*p*CO_2_), bicarbonate concentration (HCO_3^−^_) and aragonite saturation state of seawater (Ω_arag_) were calculated with CO2SYS using salinity, temperature, pH, and total alkalinity (A_T_). Values are given as mean ± SD.

Treatment	Temperature (°C)	pH (Total Scale)	Oxygen Levels (mgO_2_ L^−1^)	A_T_ (µmol Kg^−1^SW)	C_T_ (µmol Kg^−1^SW)	*p*CO_2_ (µatm)	HCO_3^−^_ (µmol Kg^−1^)	Ω_arag_
**Control (C)**	18.25 ± 0.22	8.07 ± 0.03	7.66 ± 0.033	2652.05 ± 129.84	2379.48 ± 123.82	430.52 ± 48.98	2148.33 ± 121.02	3.35 ± 0.13
**Acidification (A)**	18.10 ± 0.13	7.73 ± 0.01	7.69 ± 0.05	2712.58 ± 130.24	1063.66 ± 52.67	1063.66 ± 52.67	2483.08 ± 111.71	1.73 ± 0.07
**Deoxygenation (D)**	18.10 ± 0.11	8.07 ± 0.03	3.00 ± 0.08	2656.75 ± 120.57	2370.05 ± 120.92	429.40 ± 49.41	2140.96 ± 117.41	3.31 ± 0.19
**Warming (W)**	22.07 ± 0.08	8.06 ± 0.03	7.55 ± 0.19	2655.34 ± 129.74	2331.53 ± 122.08	432.59 ± 51.67	2080.51 ± 119.07	3.72 ± 0.17
**A + D**	18.35 ± 0.19	7.72 ± 0.01	3.01 ± 0.09	2640.03 ± 120.41	2520.63 ± 116.58	1038.35 ± 48.98	2375.59 ± 109.69	1.67 ± 0.08
**W + A**	22.07 ± 0.12	7.73 ± 0.01	7.57 ± 0.20	2704.13 ± 130.38	2551.64 ± 116.58	1059.43 ± 53.70	2391.01 ± 109.37	2.01 ± 0.09
**W + D**	22.15 ± 0.21	8.07 ± 0.03	2.95 ± 0.06	2645.64 ± 131.39	2320.55 ±131.64	423.27 ± 50.53	2066.23 ± 126.58	3.78 ± 0.16
**W + A + D**	22.02 ± 0.15	7.73 ± 0.01	2.91 ± 0.05	2738.38 ± 123.15	2593.11 ± 118.39	1086.03 ± 48.12	2430.84 ± 110.83	2.02 ± 0.10

## Data Availability

The raw data supporting the conclusions of this article will be made available by the authors on request.

## Supplementary Materials

The following supporting information can be downloaded at: https://www.mdpi.com/article/10.3390/biology15131068/s1, Table S1: Results of the statistical models applied for analysis of warming, acidification, and deoxygenation effects in the early development of *Sparus aurata*. Codes: *** when *p*-value < 0; ** when *p*-value < 0.001; * when *p*-value < 0.05; when *p*-value < 0.1. Table S2: Results of the random effect analysis of the statistical models applied for analysis of warming, acidification, and deoxygenation effects in the early development of *Sparus aurata*. Codes: *** when *p*-value < 0; ** when *p*-value < 0.001; * when *p*-value < 0.05; when *p*-value < 0.1. For Gaussian models, residual variance was estimated explicitly. For binomial GLMMs, residual variance is fixed by the distribution and not separately estimated.
