# Supplementary material for: Impacts of Warming, Acidification, and Deoxygenation on Embryos and Larvae of Gilthead Seabream (Sparus aurata)
Source: Biology (Basel). 2026 Jul 3;15(13):1068. doi: 10.3390/biology15131068 (PMC13359689; doi:10.3390/biology15131068)
Supplement: Supplementary file 1 [file biology-15-01068-s001.zip › biology-4329506-supplementary.pdf]

Supplementary Table S1. Results of the statistical models applied for analysis of warming, acidification, and deoxygenation effects in the early development of *Sparus aurata*. Codes: *** when p-value < 0; ** when p-value < 0.001; * when p-value < 0.05; . when p-value < 0.1.

	**Model**	**Terms**	**Estimate**	**Std. error**	**z value**	**Pr (>|z|)**	
Hatching success	GLMM, family=binomial	Intercept	1.359	0.492	2.761	0.0057	**
		Acidification (A)	-0.980	0.581	-1.688	0.0915	.
		Warming (W)	-0.980	0.581	-1.687	0.0915	.
		Deoxygenation (D)	-2.273	0.626	-3.631	0.0003	***
		WxA	0.627	0.793	0.790	0.4294	
		AxD	1.186	0.818	1.451	0.1467	
		WxD	1.089	0.824	1.32	0.1863	
		WxAxD	-1.763	1.167	-1.511	0.1309	
Survival	GLMM, family=binomial	Intercept	1.153	0.132	8.744	< 2e-16	***
		Acidification (A)	-0.415	0.155	-2.679	0.0074	**
		Warming (W)	-0,786	0.152	-5.191	2.09e-07	***
		Deoxygenation (D)	-1.540	0.152	-10.159	< 2e-16	***
		WxA	0.405	0.209	1.937	0.0527	.
		AxD	1.6517	0.213	7.763	8.29e-15	***
		WxD	-0.440	0.224	-1.969	0.0489	*
		WxAxD	-2.230	0.329	-6.766	1.32e-11	***
Deformities rates	GLMM, family=binomial	Intercept	-3.367	1.017	-3.311	0.0009	***
		Acidification (A)	2.39	1.124	2.123	0.0338	*
		Warming (W)	0.728	1.253	0.581	0.5611	
		Deoxygenation (D)	3.144	1.088	2.889	0.0039	**
		WxA	-2.8829	1.686	-1.710	0.0873	.
		AxD	-2.569	1.246	-2.062	0.0392	*
		WxD	-3.144	1.502	-2.093	0.0363	*
		WxAxD	5.158	1.952	2.642	0.0082	**
	**Model**	**Terms**	**Estimate**	**Std. error**	**t value**	**Pr (>|t|)**	
Heart rates	GLMM, family=Gaussian	Intercept	105.848	3.129	33.828	< 2e-16	***
		Acidification (A)	-22.197	4.526	-4.904	2.51e-06	***
		Warming (W)	31.777	4.399	7.224	2.76e-11	***
		Deoxygenation (D)	-33.754	4.845	-6.966	1.08e-10	***
		WxA	-3.628	6.963	-0.521	0.6031	
		AxD	39.565	7.362	5.374	3.02e-07	***
		WxD	-27.053	7.632	-3.545	0.0005	***
		WxAxD	-26.540	10.707	-2.479	0.0143	*
Response rates	GLMM, family=binomial	Intercept	2.124	1.093	1.944	0.0519	.
		Acidification (A)	-1.895	1.269	-1.493	0.1353	
		Warming (W)	-1.895	1.269	-1.493	0.1353	
		Deoxygenation (D)	-2.354	1.269	-1.853	0.0636	.
		WxA	1.895	1.587	1.190	0.2325	
		AxD	1.025	1.656	0.618	0.5360	

		WxD	2.354	1.587	1.477	0.1381	
		WxAxD	-20.938	6209.004	-0.003	0.9973	
Phototactic response	GLMM, family=Gaussian	Intercept	1.00e+00	1.93e-01	5.169	9.72e-01	***
		Acidification (A)	-6.67e-01	2.68e-01	-2.493	0.0258	*
		Warming (W)	-2.04e-15	2.68e-01	0.000	1.0000	
		Deoxygenation (D)	-5.00e-01	2.68e-01	-1.869	0.0826	
		WxA	4.44e-01	3.78e-01	1.175	0.2596	
		AxD	3.33e-01	3.78e-01	0.881	0.3931	
		WxD	1.67e-01	3.78e-01	0.441	0.6662	

Supplementary Table S2. Results of the random effect analysis of the statistical models applied for analysis of warming, acidification, and deoxygenation effects in the early development of *Sparus aurata*. Codes: *** when p-value < 0; ** when p-value < 0.001; * when p-value < 0.05; . when p-value < 0.1. For Gaussian models, residual variance was estimated explicitly. For binomial GLMMs, residual variance is fixed by the distribution and not separately estimated.

	**Groups**	**Name**	**Variance**	**Std. Dev.**
Hatching success	replicate	(Intercept)	0.08643	0.294
	Number of observations: 24, groups: replicate, 3			
Survival	replicate	(Intercept)	0.0013	0.114
	Number of observations: 24, groups: replicate, 3			
Deformities rates	replicate	(Intercept)	0	0
	Number of observations: 24, groups: replicate, 3			
Heart rates	replicate	(Intercept)	3.884	1.971
	residual		237.518	15.412
	Number of observations: 152, groups: replicate, 3			
Response rates	replicate	(Intercept)	0.1161	0.3407
	Number of observations: 24, groups: replicate, 3			
Phototactic rates	replicate	(Intercept)	0.00496	0.07043
	residual		0.10731	0.32758
	Number of observations: 24, groups: replicate, 3			
